# Central metabolism of functionally heterogeneous mesenchymal stromal cells

**DOI:** 10.1038/s41598-019-51937-9

**Published:** 2019-10-28

**Authors:** Mario Barilani, Roberta Palorini, Giuseppina Votta, Roberta Piras, Giuseppe Buono, Michela Grassi, Valentina Bollati, Ferdinando Chiaradonna, Lorenza Lazzari

**Affiliations:** 1 Laboratory of Regenerative Medicine – Cell Factory, Department of Tranfusion Medicine and Hematology, Fondazione IRCCS Ca’ Granda Ospedale Maggiore Policlinico, Via F. Sforza 35, 20122 Milano, MI Italy; 2 EPIGET laboratory, DISCCO department, University of Milan, Via Festa del Perdono 7, 20122 Milano, MI Italy; 3 Department of Biotechnology and Biosciences, University of Milano-Bicocca, Milan, 20126 Italy; 4 Joint Research Unit ISBE-IT Institute of Molecular Bioimaging and Physiology, National Research Council (IBFM-CNR), Segrate, MI Italia; 5 Laboratory of Transplant Immunology, UOC Transplant Coordination, Fondazione IRCCS Ca’ Granda Ospedale Maggiore Policlinico, Via F. Sforza 35, 20122 Milano, MI Italy

**Keywords:** Cell growth, Stem-cell research

## Abstract

Metabolism and mitochondrial biology have gained a prominent role as determinants of stem cell fate and function. In the context of regenerative medicine, innovative parameters predictive of therapeutic efficacy could be drawn from the association of metabolic or mitochondrial parameters to different degrees of stemness and differentiation potentials. Herein, this possibility was addressed in human mesenchymal stromal/stem cells (hMSC) previously shown to differ in lifespan and telomere length. First, these hMSC were shown to possess significantly distinct proliferation rate, senescence status and differentiation capacity. More potential hMSC were associated to higher mitochondrial (mt) DNA copy number and lower mtDNA methylation. In addition, they showed higher expression levels of oxidative phosphorylation subunits. Consistently, they exhibited higher coupled oxygen consumption rate and lower transcription of glycolysis-related genes, glucose consumption and lactate production. All these data pointed at oxidative phosphorylation-based central metabolism as a feature of higher stemness-associated hMSC phenotypes. Consistently, reduction of mitochondrial activity by complex I and III inhibitors in higher stemness-associated hMSC triggered senescence. Finally, functionally higher stemness-associated hMSC showed metabolic plasticity when challenged by glucose or glutamine shortage, which mimic bioenergetics switches that hMSC must undergo after transplantation or during self-renewal and differentiation. Altogether, these results hint at metabolic and mitochondrial parameters that could be implemented to identify stem cells endowed with superior growth and differentiation potential.

## Introduction

In the regenerative medicine field, the selection of the most appropriate stem cell type for cell therapy approaches is of paramount importance. Admittedly, inconsistencies in clinical outcomes and efficacies have been hampering a swift and straightforward translation to the clinic of promising pre-clinical stem cell-based strategies^1–3^. Specifically, discrepant potentials, poor cell engraftment and unsatisfactory survival after transplantation are enlisted among the major hurdles faced by human mesenchymal stromal/stem cells (hMSCs)^4^. Donor-to-donor heterogeneity and lack of supporting data were also indicated as issues to address and carefully re-evaluate in hMSC therapy strategies^5,6^. In this frame, an urgent need for common and standardized guidelines for clinical study design was underlined^7^. Finally, moderate and non-durable therapeutic effects were ascribed to stochastic approaches in the isolation and culture of hMSC, leading to the expansion of heterogeneous populations of cells^8^. Thus, a more precise definition of stem cell potency parameters would greatly help in the selection of stem cell champions to improve overall clinical outcomes.

In this context, metabolism is gaining more and more attention, because many reports are demonstrating that stem cell properties are directly controlled by metabolic pathways and are dependent on the capacity to switch between different bioenergetics programs^9–11^. For instance, clinical efficacy of hMSC transplantation is associated to hMSC survival in glucose shortage directly depending on their ability to adapt glycolysis in using intracellular energy reserves more than glucose^12^.

Human cord blood (hCB) offers the ideal model to address what biological parameters, including the metabolic ones, can serve as definitive read-outs to screen for best performing stem cells in clinical settings. This is because hCB is a source of different stromal/stem cell populations endowed with distinct growth and differentiation properties^13–18^. Indeed, hCB may give rise to long living (LL)- and short living (SL)-CBMSC, the former characterized by higher lifespan and differentiation properties, the latter showing earlier senescence entry. Therefore, the bioenergetics study of these two hMSC populations, unbiased by different tissue sources and donor age, is an optimal experimental setting to reveal crucial metabolic parameters potentially predictive of hMSC potential and therapeutic efficacy.

## Results

### Cord blood mesenchymal stem cells show heterogeneity in their proliferation rate, senescence status and differentiation ability

**Figure 1:**
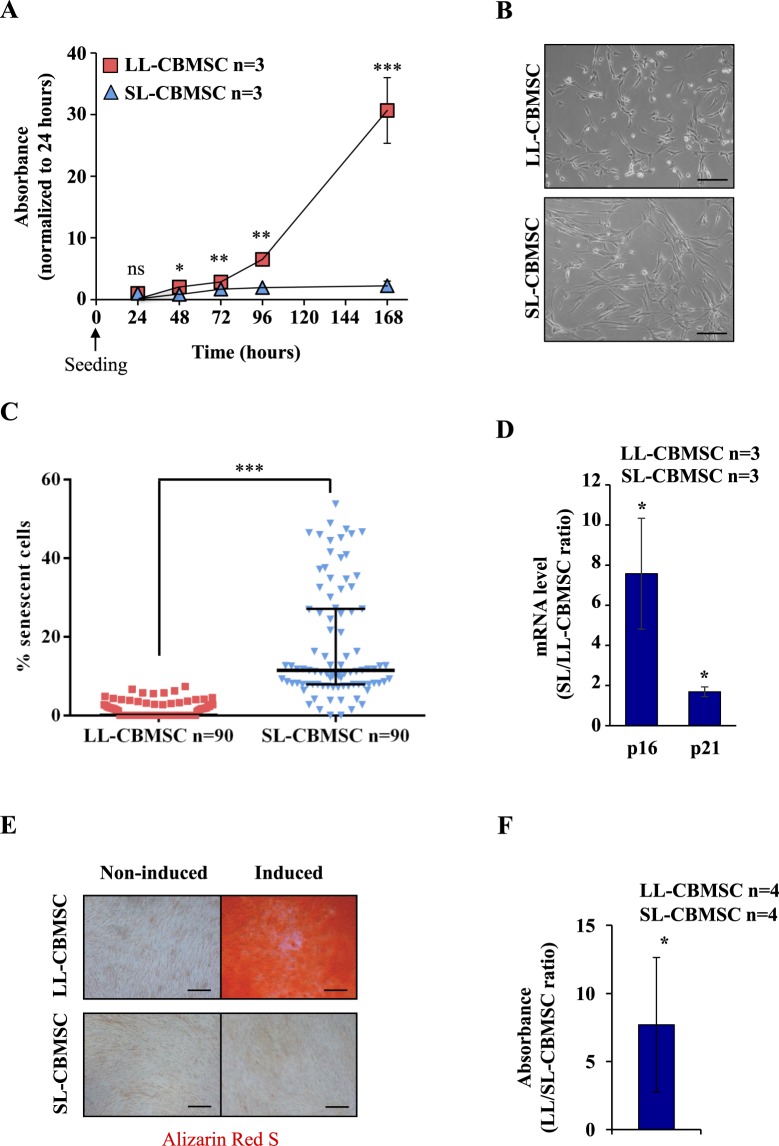
Cord blood harbours two mesenchymal stem cell populations with distinct properties. LL- and SL-CBMSC showed divergent proliferation rates measured by MTT assay (**A**). Mean and standard deviation are represented; ns, not statistically significant; *p < 0.05, **p < 0.01, ***p < 0.001 (Student’s t-test, SL-CBMSC vs. LL-CBMSC at different time points). Differences were also noted for the morphology, as shown in the bright filed images (**B**) and for senescence, as shown in β-galactosidase-positive cells quantification (**C**). Median and interquantile range are represented; ***p < 0.001 (Student’s t-test, SL-CBMSC vs. LL-CBMSC). Senescence was confirmed by p16 and p21 gene expression (**D**). Mean and standard deviation are represented; *p < 0.05 (Student’s t-test, SL-CBMSC vs. LL-CBMSC). (**E**) Different osteogenic potential was determined by Alizarin Red S staining of calcium deposits generated upon differentiation stimuli. (**F**) The amount of Alizarin Red S incorporated was biochemically quantified. Mean and standard deviation are represented; *p < 0.05 (Mann-Whitney test, SL-CBMSC vs. LL-CBMSC). Scale bar for 1B and 1E is 200 µm.

Cord blood mesenchymal stromal/stem cells (CBMSC), isolated and expanded in culture as previously described^13^, comprise two different cell populations characterized by some specific features. In particular they show marked differences in lifespan, maximum population doublings and early passage (P0-1) telomere length^13,14^. These features allowed to define a long-living (LL) population (LL-CBMSC) and a short-living (SL) population (SL-CBMSC). Herein, late passage (P5) proliferation rate and telomere length were addressed. Notable differences in proliferation rate were observed (Fig. 1A). During the exponential growth phase, LL-CBMSC showed a population doubling time (PDT) of 11 ± 0.5 hours, whereas SL-CBMSC PDT was of 24 ± 0.8 hours. Late passage LL-CBMSC telomere length was not statistical different compared to SL-CBMSC, similarly to results by previous studies^13,14^ (Supplementary Fig. 1A). This phenomenon may be ascribed to shortening of telomere sequences driven by DNA replication of highly proliferative cells (LL-CBMSC) in the absence of telomerase activity (data not shown).

The two populations were also characterized by a different morphology: SL-CBMSC possessed an enlarged size as compared to LL-CBMSC that appeared to have a thinner cell shape (Fig. 1B). Latter features correlated with a healthier and non-senescent phenotype, in line with the higher proliferation rate of LL-CBMSC in culture. To address the senescence status of LL- and SL-CBMSC, a β-galactosidase assay was performed. SL-CBMSC showed a significant higher percentage of senescent cells than LL-CBMSC (Fig. 1C and Supplementary Fig. 1B). To confirm that SL-CBMSC were more senescent, the expression of p16 and p21 was addressed by Real Time semi-quantitative PCR (qPCR) and both cell cycle regulators were found to be more expressed in SL-CBMSC than LL-CBMSC (Fig. 1D). To investigate if CBMSC with different senescence status could be isolated from the same CB unit, cord blood P0 stromal cultures were analyzed in terms of morphology and β-galactosidase positivity. As shown in Supplementary Fig. 1C P0 stromal cultures appeared composed by high proliferative and thin CBMSC forming big colonies and non-proliferative and flat CBMSC forming small colonies. Notably, colonies of proliferative CBMSC were mainly β-galactosidase-negative cells, while small colonies of non-proliferative CBMSC were all senescent, indicating that senescence is an early marker of some CBMSC (Supplementary Fig. 1D). To exclude that the more senescent phenotype of SL-CBMSC was indicative of a maternal origin, due to maternal-fetal contamination events^19–21^, short tandem repeat (STR) genetic analysis was performed. The STR profile of SL-CBMSC (n = 6) and LL-CBMSC (n = 6) was addressed and compared with that of DNA obtained from the respective mothers. In all CBMSC DNA, the expected haplotype segregation from the mother was confirmed and no maternal contamination was detected (Supplementary Fig. 1E).

To address differentiation ability, we induced CBMSC to differentiate into mesodermal mature cells *in vitro*. Our results showed that only LL-CBMSC were able to differentiate into osteocyte-like cells, whereas SL-CBMSC showed reduced or no osteogenic potential (Fig. 1E). The production of calcium deposits (mineralization) was biochemically quantified and resulted to be significantly higher in LL-CBMSC (Fig. 1F). Chondrogenesis and adipogenesis were also addressed. LL-CBMSC showed higher chondrogenic potential than SL-CBMSC (Supplementary Fig. 2A), whereas they presented similar adipogenic potential (Supplementary Fig. 2B). Altogether these findings indicate that LL-CBMSC retain higher differentiation capacity than SL-CBMSC, making the former better candidates for regenerative medicine applications.

### Mitochondrial parameters distinguish LL-CBMSC from SL-CBMSC

An important issue to address in the selection of the more appropriate hMSC to be used for further manipulation is the prediction of their cultural outcome. For this reason we decided to further characterize both cell populations for other molecular features at early passages. Several reports indicate a relation between senescence and the mitochondrial function^22–24^. Indeed, the loss of telomeres that occurs with increasing age of the cells or individuals has been associated with a decline in mitochondrial function^25^. To determine if mitochondrial features of LL-CBMSC and SL-CBMSC at the time of isolation (passage 1, P1) could be predictive of cell culture outcome, we measured mitochondrial DNA copy number (mtDNAcn), mitochondrial and global DNA methylation in parallel with the analysis of DNA oxidative stress-related modifications.

**Figure 2:**
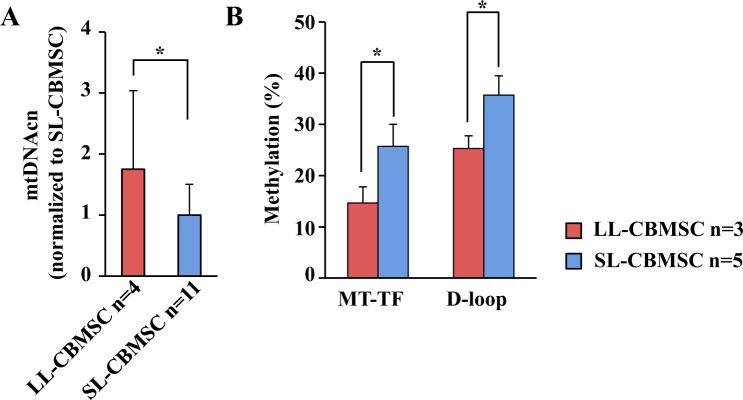
Mitochondrial DNA copy number and methylation are molecular markers of LL- or SL-CBMSC identity. (**A**) LL-CBMSC showed high mtDNAcn compared to SL-CBMSC. Mean and standard deviation are represented; *p < 0.05 (Mann-Whitney test, SL-CBMSC vs. LL-CBMSC). (**B**) LL-CBMSC also showed reduced mtDNA methylation at two sites: phenylalanine tRNA coding region (MT-TF) and mitochondrial regulatory region (D-loop). Mean and standard deviation are represented; *p < 0.05 (Student’s t-test, SL-CBMSC vs. LL-CBMSC).

As shown in Fig. 2A, LL-CBMSC mtDNAcn was two-fold higher than in the SL-CBMSC population at P1. In association with this result, LL-CBMSC showed decreased mtDNA methylation, measured at two sites, compared to SL-CBMSC (Fig. 2B), whereas equal global DNA methylation levels, estimated by LINE and SINE repeated sequences methylation, were observed (Supplementary Fig. 3A). Given that DNA methylation may be influenced by oxidative DNA damage, the 8-hydroxy-2′-deoxyguanosine (8-OHdG) cell level by qPCR^26,27^ and single and double-strand DNA breaks by single cell-gel electrophoresis (SCGE)^28^ were also evaluated. As shown in Supplementary Fig. 3B,C, both assays indicated no differences in oxidative DNA damage between LL-CBMSC and SL-CBMSC.

To further study the correlation between mitochondrial health and stemness properties in both LL-CBMSC and SL-CBMSC at later passages (P4-5), reached after expansion necessary for clinical applications, mtDNAcn, oxidative phosphorylation (OXPHOS) subunit expression, mitochondrial respiration, mitochondrial membrane potential (ΔΨm) and intracellular reactive oxygen species (ROS) were also measured.

**Figure 3:**
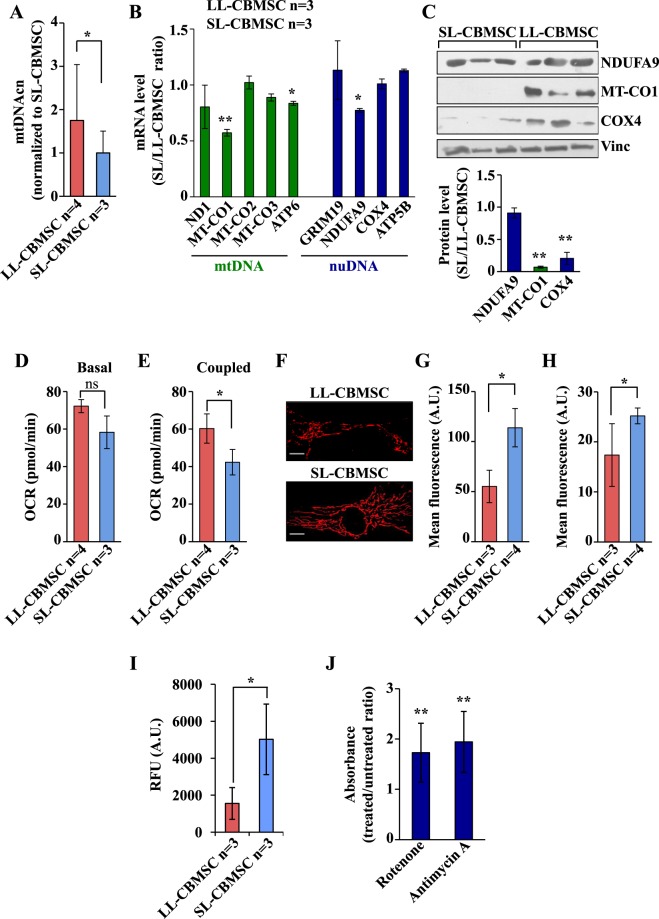
SL-CBMSC as compared to LL-CBMSC show a decrease of mitochondrial OXPHOS expression and function associated to higher mitochondrial potential. Cells were cultured for 48 hours in standard growth condition and then analyzed. (**A**) LL-CBMSC showed higher mtDNAcn, assessed by qPCR. Mean and standard deviation are represented. (**B**) qPCR analysis of the expression levels of the indicated mRNA, codified by mitochondrial DNA (mtDNA) and nuclear DNA (nuDNA). Actin was used as housekeeping gene to normalize the expression levels. (**C**) Western blot analysis of the expression levels of the indicated mitochondrial proteins. Vinculin was used to normalize the expression levels. Blots shown are derived from multiple gels run under the same experimental conditions. The membrane was cut based on molecular weight. All full length blots are presented in supplementary Fig. 4A. (**D**,**E**) Basal (**D**) and ATP production-coupled (**E**) oxygen consumption rate (OCR). The latter was calculated subtracting the OCR upon oligomycin to the basal OCR. Data are represented as mean ± SEM of the different cell populations. For each cell population n ≥ 2 biological analyses were performed with n ≥ 5 replicates for each. (**F**) Representative confocal microscopic images after staining with TMRE (50 nM). Scale bar 20 µm. (**G**-**H**) Quantification of TMRE signal using confocal microscopy (**G**) and flow cytometry (**H**). Mean and standard deviation are represented. For panels B-H, ns, not statistically significant; *p < 0.05, **p < 0.01, (Student’s t-test, SL-CBMSC vs. LL-CBMSC). (**I**) ROS levels were addressed by DHE staining and represented as relative fluorescence units (RFU) measured at 635 nm. Mean and standard deviation are represented; *p < 0.05 (Mann-Whitney test, SL-CBMSC vs. LL-CBMSC). (**J**) Senescence induced in LL-CBMSC treated by inhibitors of complex I (rotenone) and complex III (antimycin A) was detected by β-galactosidase assay and quantified as 650 nm absorbance. Mean and standard deviation are represented; **p < 0.01 (Mann-Whitney test, treated vs. untreated LL-CBMSC).

The analysis of mtDNAcn revealed that LL-CBMSC maintained also at later passages (P4-5) high mtDNAcn as compared to SL-CBMSC (Fig. 3A). However, mRNA expression analysis of five OXPHOS transcripts - *ND1* (complex I), *MT-CO1, MT-CO2* and *MT-CO3* (complex IV) and *ATP6* (complex V) - indicated that only two out of five, *MT-CO1* and *ATP6*, showed lower expression in SL-CBMSC (Fig. 3B), suggesting that the decrease in mtDNAcn as well as the increase in mtDNA methylation was not associated to a whole decrease of the mitochondrial transcripts. Nevertheless, also one out of four analyzed nuclear genes encoding for mitochondrial subunits showed lower expression in SL-CBMSC, namely NDUFA9 (complex I). Protein expression analysis confirmed the lower level of NDUFA9 and MT-CO1 in SL-CBMSC and indicated that in these cells translational and/or post-translational mechanisms could participate to regulation of mitochondrial function given that COX4 protein expression was strongly down-regulated regardless to mRNA level (Fig. 3C and supplementary Fig. 4A). Since SL-CBMSC showed a lower level of different OXPHOS transcripts and proteins, next we tried to correlate gene expression analysis with mitochondrial function by evaluating the mitochondrial respiration in both cell populations. The mitochondrial activity was estimated by measuring the oxygen consumption rate (OCR) using the XF24 Seahorse instrument. While the basal respiration was quite similar between LL- and SL-CBMSC (Fig. 3D), latter cells showed a significant reduction of the ATP-coupled OCR as compared to LL-CBMSC (Fig. 3E). To further delineate the mitochondrial function, we also measured the mitochondrial potential (ΔΨm), either by confocal microscopy (Fig. 3F,G) or flow cytometry analysis (Fig. 3H) by using the mitochondrial potential-sensitive dye tetramethylrhodamine (TMRE). Both approaches indicated that SL-CBMSC had a significant higher ΔΨm than LL-CBMSC. Since it has been shown that mitochondria that have low respiration and a high membrane potential are more prone to reactive oxygen species (ROS) generation due to proton leakage^29,30^, intracellular ROS levels were determined by dihydroethidium staining. Consistent with previous results, SL-CBMSC showed higher ROS levels than LL-CBMSC (Fig. 3I). Altogether these findings suggest the presence of less healthy mitochondria in SL-CBMSC as compared to LL-CBMSC. SCGE analysis was repeated at late passages (P4-5), yet, notwithstanding higher ROS levels in SL-CBMSC, neither LL-CBMSC nor SL-CBMSC showed the typical comet-like morphology indicative of oxidative stress-associated DNA damage (supplementary Fig. 4C).

To demonstrate a mechanistic relation between less healthy mitochondria and senescence, specifically observed in SL-CBMSC, LL-CBMSC were treated with complex I (rotenone) or complex III (antimycin A) inhibitors and assessed for their senescence by β-galactosidase assay. LL-CBMSC showed a relevant increase of senescent cells upon both rotenone and antimycin A treatment (Fig. 3J and Supplementary Fig. 4D). The effect of the two inhibitors was similar and significant. These results are in line with previous studies pinpointing mitochondrial dysfunction as a feature and a possible cause of senescence with a relevant role of complex I as a major source of intracellular ROS associated with senescence^31,32^.

Altogether these findings suggest that short-lived SL-CBMSC as compared to long-lived LL-CBMSC, in association with a reduction of proliferation and differentiation potential, show also a decline of the mitochondrial function that may directly participate in the induction of CBMSC senescence i.e. by increasing intracellular ROS.

### SL-CBMSC show increased glycolysis as compared to LL-CBMSC

**Figure 4:**
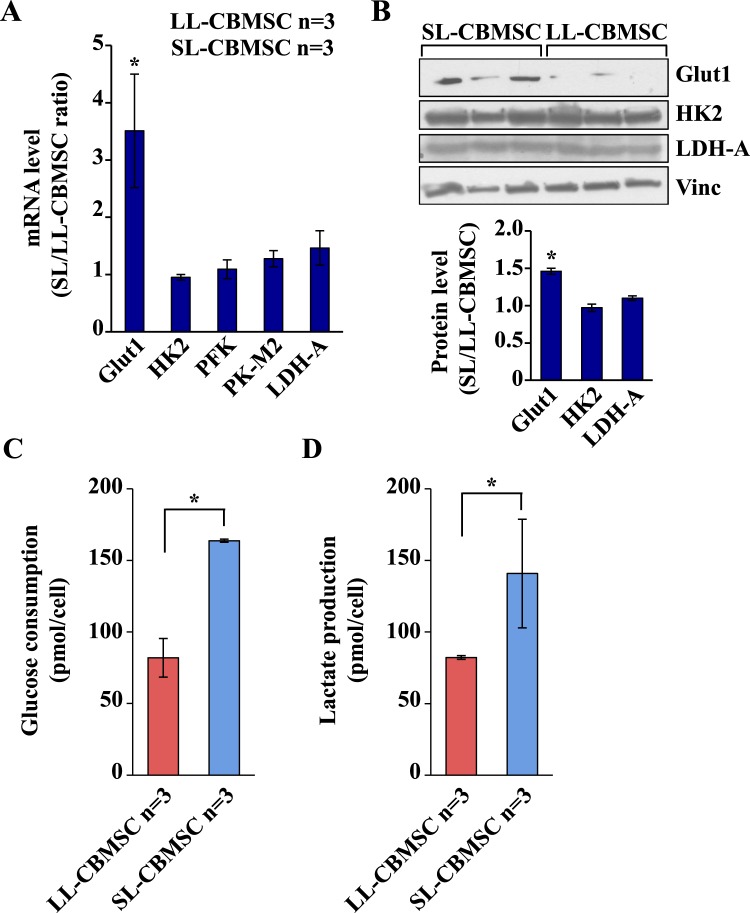
SL-CBMSC cell population shows an increased glycolytic flux. (**A**) qPCR analysis of the expression levels of the indicated mRNAs. Actin was used as housekeeping gene to normalize the expression levels. (**B**) Western blot analysis of the expression levels of the indicated glycolytic proteins. Vinculin was used to normalize the expression levels. Blots shown are derived from multiple gels run under the same experimental conditions. The membrane was cut based on molecular weight. All full length blots are presented in Supplementary Fig. 4B. (**C**) Glucose consumption and (**D**) lactate production per cell, calculated measuring the total amount of glucose and lactate in the cell media. Mean and standard deviation are represented; *p < 0.05, (Student’s t-test, SL-CBMSC vs. LL-CBMSC).

**Figure 5:**
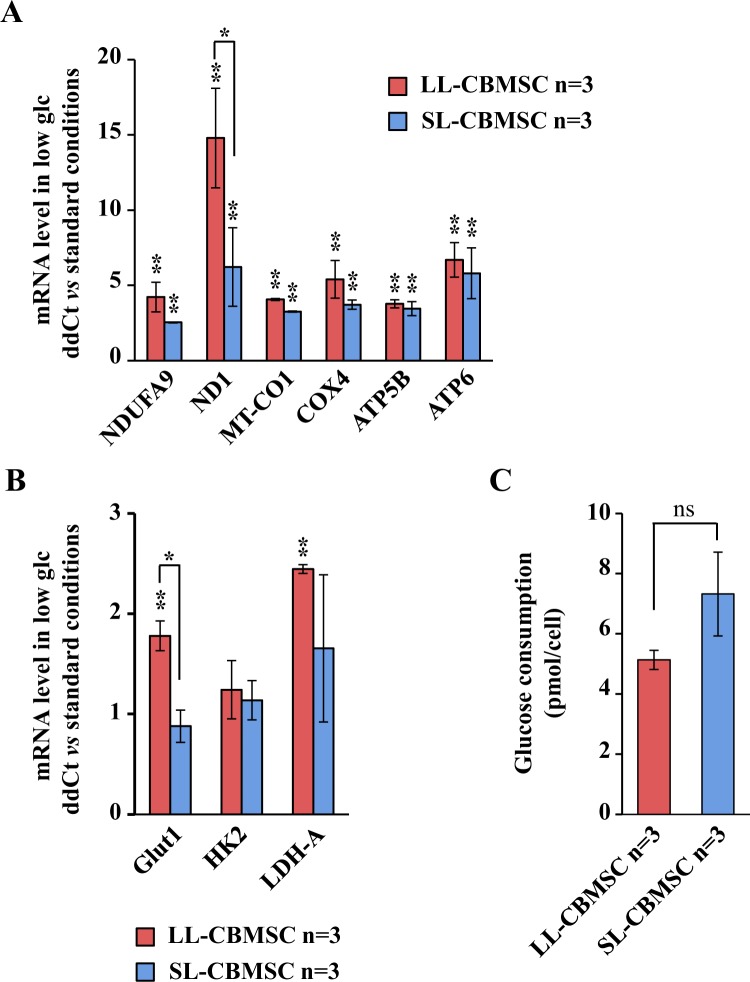
In CBMSC, particularly LL-CBMSC, glucose shortage induces the increase in expression of OXPHOS genes. CBMSC were collected and analyzed after 48 hours of growth in glucose shortage (0.5 mM glucose as initial concentration). qPCR analysis of mRNAs encoding for OXPHOS proteins (**A**) or glycolytic enzymes (**B**). (**C**) Analysis of glucose consumption. Mean and standard deviation are represented; ns, not statistically significant; *p < 0.05, **p < 0.01, (Student’s t-test; glucose shortage vs. standard condition).

A crucial property of stem cells is to modulate their metabolism in function of cell fate choices, that essentially is either to self-renew or differentiate^33^. This generally implies shifting from glycolysis to oxidative phosphorylation, moving from an undifferentiated state to a differentiated one^33^. To determine whether the different mitochondrial features observed in the two cell populations were associated with other functional changes in cellular metabolism and to a different metabolic plasticity, we next evaluated the glycolysis in both cell populations by measuring the level of mRNA expression of three glycolytic-related genes and by measuring the level of glucose consumption and lactate production. mRNA expression analysis was performed by qPCR. As shown in Fig. 4A, we evaluated the mRNA expression of the glucose transporter *Glut1* and of the mRNAs encoding for *HK2, PFK, PK-M2* and *LDH-A* enzymes. A significantly increased expression was observed only for *Glut1* in SL-CBMSC. Such a result was confirmed also by protein expression analysis (Fig. 4B and Supplementary Fig. 4B). According to this data, SL-CBMSC showed a significant increase in glucose consumption (Fig. 4C) and in lactate production (Fig. 4D). The rate of lactate secreted per glucose consumed was around 1 for both LL-CBMSC and SL-CBMSC indicating that, in both cell populations, around 50% of glucose was converted to lactate and that the glycolytic flux to the fermentative route was equal in the two populations even if in SL-CBMSC the glucose uptake was faster. To further delineate the role of glucose in both cell populations, we cultivated both in a low glucose condition shifting the cells from 25 mM glucose (normal culture condition) to 0.5 mM (low glucose condition) and analyzing their proliferation in 48 hours. As shown in Supplementary Fig. 5A,B both cell populations reduced their proliferation rate as compared to normal glucose condition. Despite such an effect on proliferation in response to glucose shortage, both highly induced mitochondrial OXPHOS mRNAs. It is worth of note that such an induction was stronger in LL-CBMSC than in SL-CBMSC (Fig. 5A) and in particular for complex I mRNAs, the major enzyme contributing to mitochondrial respiration. Indeed, complex I mRNA encoding for *NDUFA9* and *ND1* proteins showed respectively a 4-fold and 15-fold increase in LL-CBMSC as compared to 2.5 and 6-fold in SL-CBMSC. A similar higher increase in LL-CBMSC was observed also for complex IV (i.e. *MT-CO1*) as well as for glycolytic mRNAs (Fig. 5B). Notably, also in glucose shortage SL-CBMSC, although not statistically significant, showed a higher glucose uptake than LL-CBMSC (Fig. 5C). Based on these findings, we established that both populations show the ability to induce OXPHOS mRNAs under glucose shortage, but such modulation, at least for ND1 and Glut1 transcripts, appeared stronger in more proliferative and healthier LL-CBMSC. In addition, SL-CBMSC still demonstrated a trend to uptake more glucose probably to handle the reduced mitochondrial functionality.

### Glutamine is an important determinant for LL-CBMSC growth as compared to SL-CBMSC

**Figure 6:**
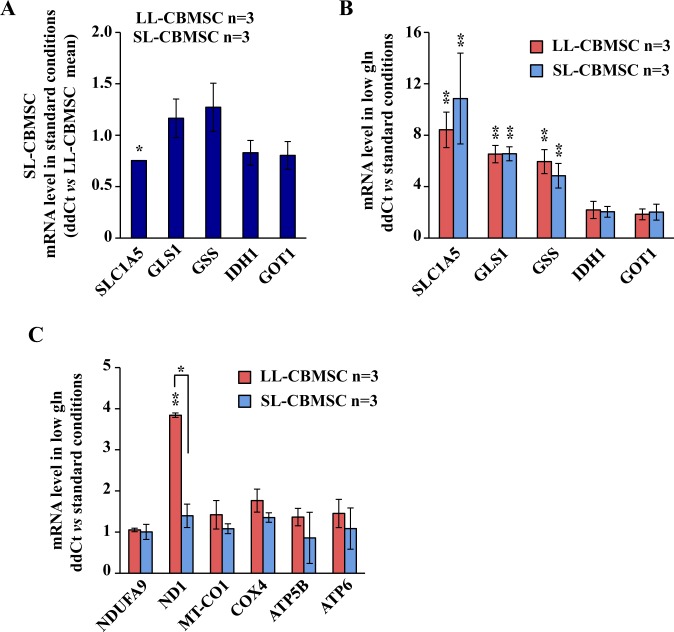
Both CBMSC populations modulate the gene expression in conditions of low glutamine. (**A**) qPCR analysis of mRNA levels in SL-CBMSC vs. LL-CBMSC grown in standard conditions. (**B**,**C**) Expression levels of mRNA encoding for glutaminolysis enzymes (**B**) and OXPHOS subunits (**C**) in glutamine shortage vs. standard conditions. Mean and standard deviation are represented; *p < 0.05, **p < 0.01, (Student’s t-test).

Previous data indicated that LL-CBMSC had a high active mitochondrial metabolism as compared to the SL-CBMSC. Since glucose shortage experiments did not significantly changed CBMSC proliferation rate, we supposed that both cell populations should also use an alternative source of energy and anabolism under normal growth condition. Thus, we decided to test the role of glutamine, another nutrient well characterized for its role in stem cell metabolism and fate^34,35^, in both proliferation and mitochondrial gene expression. To determine the role of glutamine in both cell populations, we first evaluated the mRNA expression levels of different genes involved in glutamine utilization and metabolism. In particular, we evaluated the basal levels of the main glutamine transporter *SLC1A5*, and of some enzymes involved in the anabolic routes of the glutamine such as *GLS1*, *GSS*, *IDH1* and *GOT1*. As shown in Fig. 6A, basal mRNA levels were almost similar between the two cell populations, confirming a similar ability to use glutamine in normal growth condition. Next, to better delineate the role of glutamine in both cell populations, we cultivated the cells in a low glutamine condition, shifting the culture medium glutamine from 2 mM to 0.5 mM. After 48 hours the cells were either counted or analyzed for different mRNA expression. As shown in Supplementary Fig. 6A,B, glutamine shortage induced a significant reduction in the LL-CBMSC proliferation as compared to SL-CBMSC. Indeed, LL-CBMSC proliferation was reduced of almost 56% as compared to SL-CBMSC (27% reduction). Analysis of the expression of aforementioned glutamine metabolism related mRNAs indicated that in low glutamine both cell populations increased these mRNAs significantly (between 10- and 2-fold change increase as compared to standard cell culture condition) (Fig. 6B). Conversely to glucose shortage, however, mRNAs encoding for mitochondrial electron transport chain subunits remained unchanged, except for ND1 that showed a 4-fold increase only in LL-CBMSC (Fig. 6C). These data indicate that glutamine is especially required for the proliferation and survival of LL-CBMSC suggesting that glutamine metabolism is dominant over glucose metabolism in this cell population.

## Discussion

Given the promising applications of stem cells in regenerative medicine and cell therapy^36–39^, there is increasing interest in understanding *a priori* which human mesenchymal stem cell (hMSC) population will have the best performance once transplanted. Several parameters can be considered, but recent literature has shown that first of all the metabolic aspects have to be taken into account^10,12,40–42^. To study how the metabolism can influence hMSC fate, we focused our study on two hMSC populations harvested from the same tissue source (cord blood, CB), but showing divergent properties, as demonstrated by our and other groups^13–18^. In this way, we removed any biological bias related to different donor age and tissue of origin. Our results could help in the definition of useful parameters for the selection of hMSC for more effective and consistent clinical applications. In particular, this study can be extremely informative for the regenerative medicine applications of CB, that presents many attractive advantages, including a noninvasive collection procedure, low risk of infection for the donor, nontumorigenesis, multipotency and low immunogenicity^33^.

Herein, we report that CBMSC, derived from different donors, show a clear level of intrinsic heterogeneity since they comprise at least two different cell populations, according to some recent data^43^. Importantly, we show that these two populations, characterized by a different proliferation rate, senescence status and differentiation potential, are also characterized by a distinct cell metabolism, strictly associated to a different mitochondrial function. The first evidence of such biological phenotype derives from the observation that short-living (SL)-CBMSC show a reduction of mitochondrial DNA copy number (mtDNAcn) as compared to long living (LL)-CBMSC. Most studies reported mtDNA abundance changes in relation to aging in many tissues of humans, rats or mice^44,45^ as well as in human stem cells^46^. In all these reports, sometimes with conflicting results, an association between the mtDNAcn decrease and aging has been widely described. In our case, we observed an association between lower growth potential and increased senescence of SL-CBMSC and decrease of mtDNAcn as compared to LL-CBMSC. Interestingly, despite the somehow conflicting results on the meaning and mechanisms of the mtDNA methylation^47,48^, we clearly observed an enhanced mtDNA methylation. While not always a direct correlation between the hypermethylation and mitochondrial gene and protein expression has been observed, SL-CBMSC showed lower expression of MT-CO1 and COX4 OXPHOS subunits as compared to LL-CBMSC. In particular, the down-regulation of MT-CO1 and consequently of the mitochondrial activity, has been already considered a marker of aging in *Drosophila*^49^ and in human tissues^50^. Conversely, while no association between COX4 and aging has been previously shown, its important role for complex IV assembly and OXPHOS function^51^, convincingly suggest an association between mitochondrial dysfunction and senescence, at least in SL-CBMSC. Accordingly, SL-CBMSC show a reduction of mitochondrial activity, associated to a senescent phenotype less prone to differentiation. On the other hand, SL-CBMSC showed a consistently higher mitochondrial potential (ΔΨm). This result, that was quite unexpected, is in agreement with a recent report in which it has been shown that stem cells with low ΔΨm possess more evident stem cell-like characteristics such as great self-renewal and a long-term cellular engraftment as compared to stem cells with a higher ΔΨm^52^. Moreover, alike to our results, cells with high ΔΨm show also an increased glycolysis, in accordance with what we observed for SL-CBMSC. Therefore, we can suppose that CBMSC have a similar energetic metabolism and in particular that LL-CBMSC, keeping a low energetic state (oxidative metabolism and low ΔΨm) as compared to SL-CBMSC (glycolytic metabolism and high ΔΨm), are less prone to generate reactive oxygen species (ROS), which may trigger senescence, as demonstrated by our results, but metabolic responsive under request. Indeed, analyses of SL-CBMSC and LL-CBMSC metabolic plasticity performed under an energetic stress, such as glucose and glutamine shortage, while showed almost an equal capability of both CBMSC to increase the expression of different mitochondrial mRNAs, also underlined the significant and specific increase in LL-CBMSC of the ND1 subunit, already shown as essential for the electron transport chain Complex I assembly^53,54^. This feature well fits with their great capability to differentiate, a process in which the mitochondrial function is fundamental^55^. Furthermore, although ROS exert oxidative damage on lipids, proteins, and DNA, they also act as secondary messengers and were recently shown to be involved in the regulation of stem cell self-renewal, pluripotency, and differentiation^56^. In particular, excessive ROS hamper osteogenic differentiation^57^, whereas low ROS levels preserve MSC stemness^58^. On the other hand the low level of ROS observed in LL-CBMSC may be consequent to a better glutamine utilization for anaplerosis, involving also an healthy mitochondria, such as glutathione synthesis and TCA cycle fuelling, former synthesis able to retard premature cell senescence in different cell models^59^ and to contribute to the maintenance of stem cell function^60,61^. Interestingly, LL-CBMSC being more sensitive to glutamine shortage than SL-CBMSC, suggest again a better mitochondrial functionality. Furthermore, we have also to underline that activation of glycolysis, preferred metabolic state of rapidly proliferating normal and cancer cells and less differentiated stem cells^55,62^, is also the main metabolic characteristic of senescent cells, as confirmed in many studies^63–66^, which again hints at a senescence-like phenotype for SL-CBMSC. Importantly, such a glycolytic switch may be considered consequentially to a reduced mitochondrial function more than a significant change of glycolytic genes and proteins, as we show in our SL-CBMSC.

In conclusion, we propose that different hMSC populations diverging for stemness and differentiation features can be distinguished based on their central metabolism. These metabolic characteristics may help in the selection, optimization and *in vitro* maintenance of the appropriate stem cell populations for regenerative medicine applications.

## Methods

### Cell culture

The CB-derived hMSC used in this work were previously obtained and fully characterized as described in detail in recent publications^13,14,67^. The definition of LL- and SL-CBMSC was applied retrospectively based on lifespan and growth properties as previously described (Barilani, 2015). Stored cell samples were used in the present study accordingly to that definition. Culture medium consisted of αMEM-GlutaMAX (Invitrogen, Carlsdad, CA, USA) supplemented with 20% fetal bovine serum (FBS; Invitrogen) and medium changes were performed twice a week. Cell cultures were maintained at 37 °C in a humidified atmosphere containing 5% CO_2_. At 80% confluence, the cells were harvested using 25% TrypLE Select 1× (Invitrogen) and were washed with PBS (Invitrogen) and cultured at a concentration of 4 × 10^3^ cells/cm^2^. The authors state that this study was performed according to the amended Declaration of Helsinki. In addition, written informed consent has been obtained from all the cord blood donors involved in the study and use of human tissue and cells was approved by the Ethical Committee of our institute “Fondazione IRCCS Ca’ Granda Ospedale Maggiore Policlinico”.

### Microculture tetrazolium assay

Microculture tetrazolium (MTT) assay was performed as elsewhere described^68^ with slight changes. Briefly, 4,000 cells/cm^2^ were seeded into 96-well plates in quadruplicates and at 24, 48, 72, 96, 168 hours, MTT assay was performed. A volume of 200 μL of 0.5 mg/mL Thiazolyl Blue Tetrazolium Bromide (Sigma-Aldrich, Saint Louis, MI, USA) in DMEM without phenol red (Invitrogen) were added to each well and the colorimetric reaction was allowed to proceed for 2 hours at 37 °C in the dark. Next, the solution was removed and 200 μL of 96% ethanol were added and the plate incubated 30 minutes at 37 °C in the dark. MTT dye uptake was determined by measuring the optical density at 570 nm after background subtraction scored at 650 nm, using GENios microplate reader (TECAN, Männedorf, Switzerland) and analyzing the data with Magellan software (TECAN). Population doubling time was calculated as follows: PDT = [(t2 − t1) × log(2)]/[(log(A2) − log(A1)], where t1 and t2 are two time points of exponential growth, while A1 and A2 are the respective absorbance values normalized to 24 hour time point.

### Telomere length

Telomere length was assessed by qPCR as elsewhere described^13,14^. Briefly, LL- and SL-CBMSC were collected from confluent cultures and DNA was extracted with QIAamp DNA Blood Mini Kit (51104; Qiagen, Hilden, Germany) following manufacturer’s instructions. The telomere sequence and single copy gene (36B4) specific amplification reactions were performed in triplicate in 96-well plates on a CFX96 machine (Bio-Rad, Hercules, CA, USA). The data analysis was carried out using CFX Manager software (Bio-Rad).

### β-galactosidase activity

β-galactosidase staining was performed using the Senescence Cells Histochemical Staining Kit (CS0030; Sigma-Aldrich). For analysis of P0 cells, CBMSC were obtained from buffy coat as already described^14^ and analyzed 2 weeks after the appearance of colonies and cells with CBMSC morphology. For analysis of P5 cells, LL- and SL-CBMSC were harvested from 80% confluent cultures and seeded in 3 wells of 24-well plates at 10,000 cells/cm^2^ and analyzed after 24 hours. To analyze β-galactosidase activity, both P0 and P5 cultures were washed twice with PBS (Invitrogen), fixed with 1 × Fixation Buffer (CS0030; Sigma-Aldrich) for 7 minutes at RT and washed again twice with PBS (Invitrogen). Next, the cells were incubated with X-gal-containing Staining Mixture (CS0030; Sigma-Aldrich) at 37 °C overnight and then washed with PBS (Invitrogen). Images of stained cells were acquired with a Nikon Eclipse TS100 microscope (Nikon). At P0, all colonies and single cells showing CBMSC morphology were analyzed. For P5 LL- and SL-CBMSC, 10 fields containing at least a mean of 50 cells were acquired for each well and analyzed for positive staining.

For the analysis of senescence after treatment with ETC inhibitors, LL-CBMSC cells were seeded at 2,000 or 4,000 cells/cm^2^ density and the day after they were treated with rotenone (Sigma) 50–100 nM and antimycin A (Sigma) 50 nM. The cells were fixed for the assay after 1 week. Positivity to β-galactosidase was quantified by reading the absorbance at 650 nm on a GENios microplate reader (TECAN).

### Short tandem repeats

Genomic DNA was isolated from 100,000 CBMSC and from maternal serum with the QIAamp 96 DNA Micro kit (56304; Qiagen) following manufacturer’s instructions. A total of 15 short tandem repeat (STR) loci (D5S818, vWA, FGA, D19S433, TPOX, D16S539, D3S1358, TH01, D2S1338, D8S1179, D21S11, D18S51, D13S317, D7S820, CSF1PO) and a segment of the X-Y homologous gene Amelogenin were amplified using the AmpFlSTR Identifiler Plus kit (4427368; Applied Biosystems, Foster City, CA, USA) according to the manufacturer’s instructions. Electrophoretic analysis was carried out on an ABI PRISM 3130 Genetic Analyzer (Applied Biosystems) using POP-7 Polymer for 3130 Genetic analyzer (Applied Biosystems). The length of amplified DNA fragments was determined in comparison with GeneScan-500 LIZ internal size standard (4322682; Applied Biosystems). Each electrophoretic run was analyzed with GeneScan Analysis software (Applied Biosystems).

### Osteogenesis, chondrogenesis and adipogenesis

Osteogenic induction medium (PT-3002; Lonza, Basel, Switzerland) was used to promote osteogenesis, as elsewhere described (Barilani, 2015; Barilani, 2016). Briefly, 2 × 10^4^ LL- (n = 3) or SL-CBMSC (n = 3)/cm^2^ were plated in αMEM-GlutaMAX (Invitrogen) supplemented with 20% FBS (Invitrogen); the day after the medium was switched to differentiation medium. The cells were maintained in culture for 2 weeks, with three medium changes per week. Calcium deposits were stained with Alizarin Red S (A5533; Sigma-Aldrich): the cells were washed with PBS (Invitrogen), fixed for 30 minutes at 4 °C with 70% ethanol (02870-1L-F; Sigma-Aldrich), washed with PBS (Invitrogen), stained for 15 minutes at RT with 2% Alizarin Red S solution, and finally multiple washing steps were performed with PBS (Invitrogen). Images of calcium deposits were acquired on a Nikon Eclipse TS100 microscope (Nikon, Tokyo, Japan). For the biochemical quantification of calcium deposits, the cultures were incubated with 10% acetic acid for 1 hour at RT to extract Alizarin Red S staining. Absorbance was read at 405 nm on a microplate reader (GENios; TECAN, Männedorf, Switzerland). Induced sample absorbance values were corrected with non-induced ones and normalized on SL-CBMSC mean value. To promote chondrogenesis, chondrogenesis induction medium (PT-3003; Lonza) supplemented with 10 ng/mL TGFβ3 (PT-4124; Lonza) was used, following manufacturer’s instructions. Chondrogenic cell pellets were harvested after 3 weeks and analyzed by standard histological techniques using hematoxylin-eosin (1.05174; Sigma-Aldrich) to stain cell structures and Alcian blue (B8438; Sigma-Aldrich) to stain acidic polysaccharides-rich cartilage-like tissue. Images were acquired on a Nikon Eclipse 80i microscope (Nikon). To promote adipogenesis, adipogenesis maintenance and induction medium (PT-3004; Lonza) were used as elsewhere described (Barilani, 2015; Barilani, 2016). Lipid droplets-rich adipocyte-like cells were stained by Oil Red O (O0625; Sigma-Aldrich). Images were acquired on a Nikon Eclipse TS100 microscope (Nikon).

### Mitochondrial DNA copy number

Mitochondrial DNA copy number (mtDNAcn) was assessed by qPCR performed on a Bio-Rad CFX96 (Bio-Rad, Hercules, CA) preparing one 96-well plate for the amplification of the mitochondrial DNA regulatory region (mtMinArc) and one for the amplification of a reference nuclear single copy gene (36B4). A pool of all the samples was used to build a standard curve by 1:1 serial dilution (100–1.56 ng) and thermal reaction conditions for optimal amplification efficiency were established for each couple of primers. Every plate was loaded with the standard curve and the samples to analyze in triplicate in the respective reaction mix. The reaction components were as follows: 1× SsoFast EvaGreen Supermix (Bio-Rad), 400 nM of reference gene forward and reverse primers, or 500 nM of mtMinArc forward and reverse primers, and 12 ng of DNA in a final reaction volume of 10 μL. The thermal profile was set up for optimal activity of SsoFast EvaGreen Supermix (Bio-Rad): enzyme activation at 98 °C (2 minutes), 46 cycles of 98 °C (5 seconds), 60 °C (20 seconds) for minArc region assay plate; enzyme activation at 98 °C (2 minutes), 46 cycles of 98 °C (2 seconds), 65 °C (2 seconds) for reference gene assay plate. The analysis was carried out using Bio-Rad CFX Manager software (Bio-Rad).

### DNA methylation

500 ng DNA (concentration 25 ng/µL) was treated using EZ DNA Methylation-Gold Kit (Zymo Research, Orange, CA, USA) according to the manufacturer’s protocol. Final elution was performed with 30 µL of M-Elution Buffer. We performed DNA methylation analyses on bisulfite-treated DNA using highly quantitative analysis based on PCR-pyrosequencing. The PCR and pyrosequencing primer sequences for repetitive element subfamilies^69–71^ and mitochondrial DNA^71^ methylation analysis have been previously published. Briefly, a 50 µL PCR was carried out in 25 µL of GoTaq Green Master mix (Promega, Madison, WI, USA), 1 pmol of the forward primer, 1 pmol of the biotinylated reverse primer, 25 ng of bisulfite-treated genomic DNA and water. The biotin-labelled primers were used to purify the final PCR product using Sepharose beads. The PCR product was bound to Streptavidin Sepharose HP (Amersham Biosciences, Uppsala, Sweden) and the Sepharose beads containing the immobilized PCR product were purified, washed, denatured using a 0.2 M NaOH solution, and washed again using the Pyrosequencing Vacuum Prep Tool (Pyrosequencing, Inc., Westborough, MA), as recommended by the manufacturer. Then, 0.3 µΜ pyrosequencing primer was annealed to the purified single-stranded PCR product and pyrosequencing was performed using the PyroMark MD System (Pyrosequencing, Inc.). As a quality control check to estimate bisulfite conversion efficiency, we placed duplicate genomic DNA samples on each bisulfite conversion plate to estimate the internal plate variation of bisulfite conversion and the pyrosequencing reaction. The degree of methylation was expressed as percentage of methylated cytosines divided by the sum of methylated and unmethylated cytosines (%5mC). Every sample was tested three times for each marker to confirm reproducibility and increase precision of our results. The average of the three replicates was used in statistical analyses.

### 8-OHdG detection

Mitochondrial 8-OHdG was measured using qPCR as previously described^72,73^. For the digestion reaction, 4 μL of DNA were incubated for 1 h at 37 °C with 11 μL of reaction mix containing RNase free water (8.7 μL/reaction), buffer NE 10× (1.5 μL/reaction), BSA 100× (0.15 μL/reaction), and human oxoguanine glycosylase 1 (hOGG1) or RNase free water (0.625 μL/reaction) for the treatment and non-treatment condition, respectively. The samples were diluted 1:4 in RNase free water and a fragment of mtDNA was amplified using a master mix consisting of 1× SsoFast EvaGreen Supermix (5 μL/reaction; Bio-Rad), forward (0.5 μL/reaction) and reverse (0.5 μL/reaction) primers (F:5′-CACCCAAGAACAGGGTTTGT-3′ and R:5′-TTAACAACATACCCATGGCCA-3′), and 4 μL of treated or non-treated DNA. Samples were run in triplicate in 96-well plate compatible with the CFX96 Real-Time system (Bio-Rad). The thermal cycling profile started with 10 s at 95 °C, followed by 35 cycles of 15 s at 95 °C plus 1 min at 60 °C. Bio-Rad CFX Manager software (Bio-Rad) was used to analyze qPCR data and differences in amplification efficiency between non-treated and treated DNA (∆Ct) were calculated.

### Single cell-gel electrophoresis

Single cell-gel electrophoresis was performed in three passages: incapsulation, lysis and electrophoresis. For the incapsulation phase, 25,000 LL- or SL-CBMSC resuspended in 100 µL of 0.7% low melting-agar (LMA; Sigma-Aldrich) in PBS (Invitrogen) were spotted onto a microscope slide holder previously coated with 1% agarose (Sigma-Aldrich) in PBS (Invitrogen), subsequently covered by 0.7% LMA in PBS (Invitrogen). For the lysis phase, the samples were incubated for 1 hour at 4 °C in lysis buffer. Lysis buffer consisted of 2.5 M NaCl (Sigma-Aldrich), 100 mM disodium EDTA (Sigma-Aldrich), 10 mM Trizma base (Sigma-Aldrich), 10% v/v DMSO (Sigma-Aldrich), 0.1% v/v Triton X-100 (Sigma-Aldrich) and 200 mM NaOH (Sigma-Aldrich) in distilled water, pH = 10. For the electrophoresis phase, the samples were washed twice with running buffer for 10 minutes at 4 °C and placed in an electrophoresis apparatus filled with running buffer. Running buffer consisted of 300 mM NaOH (Sigma-Aldrich), 1 mM disodium EDTA (Sigma-Aldrich) in distilled water, pH > 13. The electrophoresis was performed at 300 mA for 20 minutes in the dark. Then, the samples were washed twice with neutralization buffer for 5 minutes in the dark at RT. Neutralization buffer consisted of 400 mM Trizma base (Sigma-Aldrich) in distilled water, pH = 7.5. To stain nuclear DNA, the samples were incubated with GelRed (Sigma-Aldrich) diluted 1:1,000 in water for 3 minutes in the dark at RT, and then washed with distilled water for 5 minutes. Images of cells forming or not the so-called comets due to the presence of single and double DNA strand breaks, apurinic sites or excision-repair sites were then taken with a Nikon Eclipse 80i microscope (Nikon) using Image-Pro Plus 6.2 (Media Cybernetics, Bethesda, MD) and the data processed with Comet Assay Software Project (CASP) 1.2.2 software (http://casplab.com/) following published methodology^74^.

### RNA extraction and semiquantitative RT-PCR analysis

RNA extraction and reverse transcription and semiquantitative PCR were performed as already described^75,76^. β-actin was used as endogenous control. Primers are the following: p16 forward TTCCCCCACTACCGTAAATG, reverse CACTCCAGAAAACTCCAACACA; p21 forward CATGTGGACCTGTCACTGTCTTGTA, reverse GAAGATCAGCCGGCGTTTG; ND1 forward CCCTGGTCAACCTCAACCTA, reverse CTAGTTCGGACTCCCCTTCG; MT-CO1 forward ACGTTGTAGCCCACTTCCAC, reverse AGCGAAGGCTTCTCAAATCA; MT-CO2 forward TAACATCTCAGACGCTCAGGAA, reverse GTTGAAGATTAGTCCGCCGTAG; MT-CO3 forward AAATCCCCTAGAAGTCCCACTC, reverse CTCTGAGGCTTGTAGGAGGGTA; ATP6 forward TATTGATCCCCACCTCCAAA, reverse GATGGCCATGGCTAGGTTTA; GRIM19 forward AGATGCTTCGGGAGAACCTG, reverse GGCCTACGTGTACCACATGA; NDUFA9 forward CAGATTGTTCCTCCCATTCC, reverse TGCATCCGCTCCACTTTATC; COX4 forward TTCGCTCCCAGCTTATATGG, reverse GCTTCTGCCACATGATAACG; ATP5B forward TTGCTATGGATGGTACAGAAGG, reverse TTTGGTTTTGATGGGACCTC; Glut1 forward CTCCCCACACACACAAAA, reverse CCAAATCGGCATCTTCTCAT; HK2 forward TAGGGCTTGAGAGCACCTGT, reverse CCACACCCACTGTCACTTTG; PFK (PFK-M) forward TGGGACTAAAAGGACTCTACCC reverse CCCTGTGTAAGCCTCAAAGC; PK-M2 forward GGGGTTCGGAGGTTTGATGAA reverse TTGCAAGTGGTAGATGGCAGC; LDHA forward TGTGCCTGTATGGAGTGGAA, reverse AGCACTCTAACCACCTGCT; SLC1A5 forward TGCGTGGAGGAGAATAATGG, reverse GGATGATGGCCAGAGTGAGGA; GLS1 forward TGGTGGCCTCAGGTGAAAAT, reverse CCAAGCTAGGTAACAGACCCTGTTT; GSS forward GGAACATCCATGTGATCCGACGA, reverse CCTTCTTAGTCCCAGCCAGCT; IDH1 forward TGGCTCTGTCTAAGGGTTGG, reverse ACCATGTCGTCGATGAGCCT; GOT1 forward GGAGCAGTGGAAGCAGATTGC, reverse AGCCCGAAGTTCTTGGAGAAGG; Actin forward GCCTTTATTGCTTCCAGCAG, reverse CGTGGATGCCACAGGACT.

### Western blot analysis

Cells were harvested and disrupted in RIPA buffer (R0278; Sigma) containing protease inhibitors (P8340; Sigma-Aldrich); 50 μg of total proteins were resolved by SDS-PAGE and transferred to the nitrocellulose membrane, which was incubated overnight with specific antibodies: Vinculin and Glut1 from Santa-Cruz Biotechnology; NDUFA9 and MT-CO1 from Boster Biological Technology; COX4 from Abcam; HK2 from Cell Signaling Technology; LDH-A from CHEMICON by EMD Millipore Corporation.

### Measurement of OCR with the Seahorse instrument

The oxygen consumption rate (OCR) was determined using the Seahorse XF24 extracellular flux analyzer (Agilent). LL- or SL-CBMSC were seeded onto wells of a dedicated 24-well XF24 cell culture plate in their standard medium. The day after, one hour before the analysis the culture medium was exchanged for a specific base medium (complete formulation: XF unbuffered DMEM supplemented with 1% FBS, 5.5 mM glucose, 1 mM sodium pyruvate and 2 mM glutamine) and the cells were incubated at 37 °C without CO_2_ before running the experiment. The basal OCR was measured three times; then, three measurements of OCR were performed after injection of 1 µM oligomycin. After the analysis the cells were counted and all OCR values obtained by the instrument were normalized on the cell number. The coupled OCR was calculated subtracting the OCR upon oligomycin (lowest measurement) to the basal OCR (third measurement).

### Mitochondrial potential

Mitochondrial potential was evacuate by staining cells with Tetramethylrhodamine ethyl ester (TMRE, Sigma-Aldrich) with both confocal microscopy and flow cytometer according to the manufacturer’s protocol with minor modifications. Briefly, for the analysis with the confocal microscopy 1 × 10^4^ LL- or SL-CBMSC/compartment were seeded in cell view cell culture dish (Greiner) and incubated under normal growth conditions. After 48 hours the cells were stained with 50 nM TMRE for 15 minutes at 37 °C. Cells were examined under a A1R Nikon laser scanning fluorescence confocal microscope at a magnification of 40× to obtain a minimum of 15 frames per field. Collected fluorescence emission was quantified using NIS-Elements AR analysis 4.10.00 software by Nikon. To measure the fluorescence, each cell in the field was considered as a separate event whose fluorescence intensity is put in mean with all events captured for the sample. Flow cytometric analysis was performed using the CytoFLEX platform (Beckman Coulter). In particular, 5 × 10^4^ cells were collected in 50 μL of binding buffer and stained with 100 nM TMRE, for 15 minutes at 37 °C. After the incubation, samples were diluted in an appropriate volume of binding buffer (10 mM HEPES/NaOH pH 7.4, 140 mM NaCl, 2.5 mM CaCl_2_) and analyzed.

### Reactive oxygen species

Dihydroethidium (DHE) was used to quantify total intracellular reactive oxygen species (ROS). LL- and SL-CBMSC grown in 24-well plates were stained for 20 minutes at 37 °C with a fresh 0.5 µM DHE working solution (0.5 mL/well) prepared in DMEM without phenol red (Invitrogen) from a 5 mM stock. The cultures were washed twice with PBS (Invitrogen) and fluorescence was read at 635 nm (excitation at 535 nm) using GENios microplate reader (TECAN) and analyzing the data with Magellan software (TECAN).

### Measurement of D-glucose and L-lactic acid

Concentrations of D-glucose and L-lactic acid in the supernatants were determined by using the D-glucose and L-Lactic acid UV-method kits (r-Biopharm- Roche, Basel, CH) according to the manufacturer’s protocol.

### Statistical analysis

All statistical analyses were performed using Prism 6 (GraphPad Software, La Jolla, CA, USA). A p-value < 0.05 was considered statistically significant.

## Supplementary information

Supplementary figures

## Data Availability

Data used to support the findings of this study are available from the corresponding author upon request.
