# Supplementary material for: Central metabolism of functionally heterogeneous mesenchymal stromal cells
Source: Sci Rep. 2019 Oct 28;9:15420. doi: 10.1038/s41598-019-51937-9 (PMC6817850; doi:10.1038/s41598-019-51937-9)
Supplement: Supplementary file 1 — Supplementary figures [file 41598_2019_51937_MOESM1_ESM.pdf]

Central metabolism of functionally heterogeneous mesenchymal stromal cells

Mario Barilani[1,2,*], Roberta Palorini[3,*], Giuseppina Votta[3,4], Roberta Piras[1], Giuseppe Buono[1], Michela Grassi[5], Valentina Bollati[2], Ferdinando Chiaradonna[3,#] and Lorenza Lazzari[1,#]

[1]Laboratory of Regenerative Medicine – Cell Factory, Department of Tranfusion Medicine and Hematology, Fondazione IRCCS Ca' Granda Ospedale Maggiore Policlinico, Via F. Sforza 35, 20122 Milano (MI), Italy
[2]EPIGET laboratory, DISCCO department, University of Milan, Via Festa del Perdono 7, 20122 Milano (MI), Italy
[3]Department of Biotechnology and Biosciences, University of Milano-Bicocca, Milan, 20126, Italy
[4]Joint Research Unit ISBE-IT Institute of Molecular Bioimaging and Physiology, National Research Council (IBFM-CNR), Segrate, MI, Italia
[5]Laboratory of Transplant Immunology, UOC Transplant Coordination, Fondazione IRCCS Ca' Granda Ospedale Maggiore Policlinico, Via F. Sforza 35, 20122 Milano (MI), Italy

* These authors contributed equally to this work
# To whom correspondence should be addressed: lorenza.lazzari@policlinico.mi.it and ferdinando.chiaradonna@unimib.it

Email addresses of all authors:
m.barilani@gmail.com; roberta.palorini@unimib.it; giuseppina.votta@unimib.it; robi.piras91@gmail.com; giuseppe.buono@policlinico.mi.it; michela.grassi@policlinico.mi.it; valentina.bollati@unimi.it; ferdinando.chiaradonna@unimib.it; lorenza.lazzari@policlinico.mi.it.

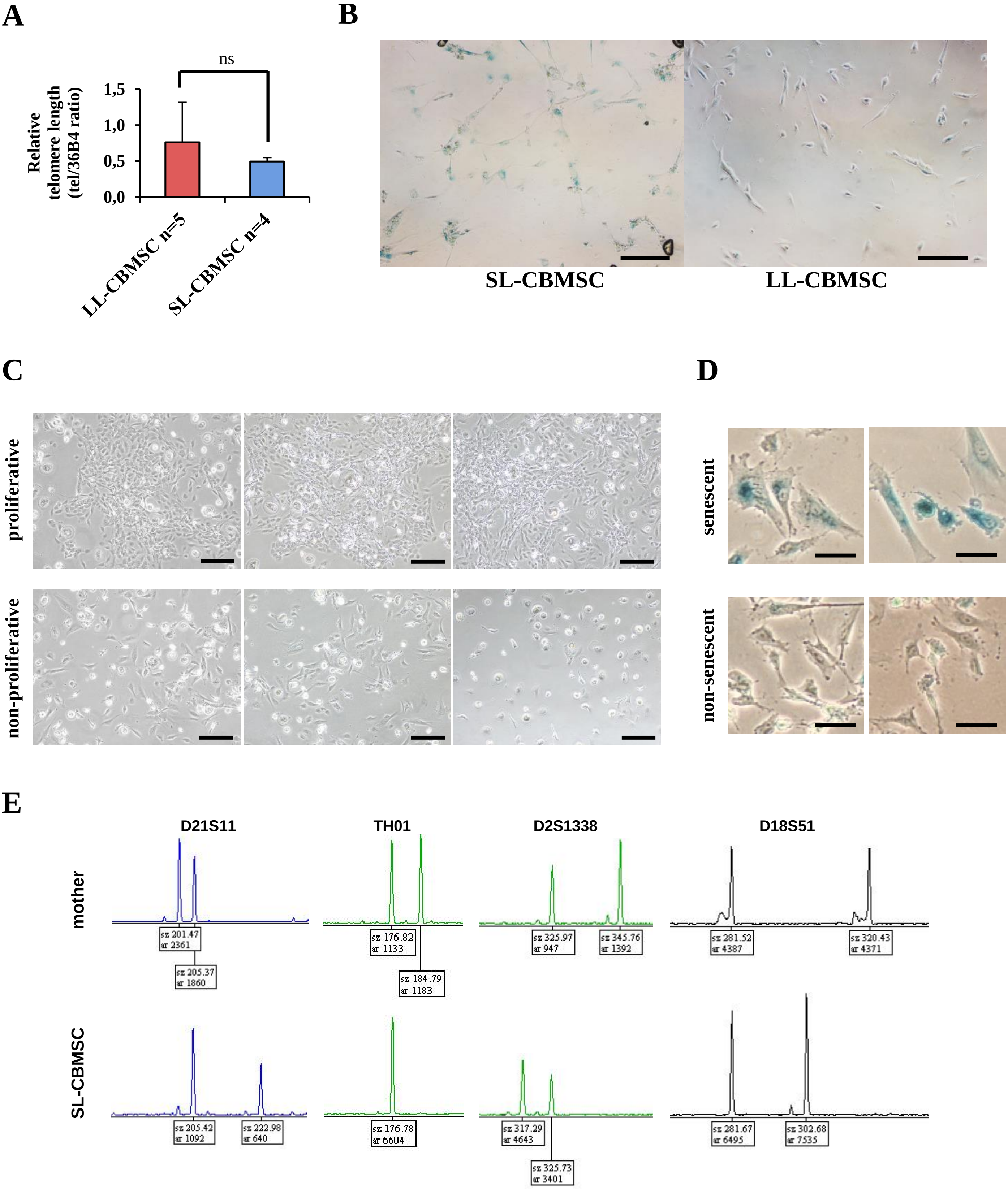

Supplementary figure 1. LL- and SL-CBMSC show similar telomere length at P5 and different senescence status in spite of absence of maternal cell contamination. Histograms show relative telomere length (A); tel, telomere sequences; 36B4, single copy gene; ns, statistically not significant (Student's t-test); mean and standard deviation are represented. Representative images of P5 SL-CBMSC and LL-CBMSC stained to assess β-galactosidase activity (B). Scale bar 200 µm. Representative images of CBMSC colonies formed by more proliferative (proliferative) or less proliferative (non-proliferative) cells at P0 (C). Scale bar 200 µm. Representative images of β-galactosidase-negative (non-senescent) or positive (senescent) CBMSC clusters (D). Scale bar 100 µm. Representative SL-CBMSC short tandem repeat profile compared to that obtained from the DNA of the mother for 4 out of 15 analyzed loci (E).

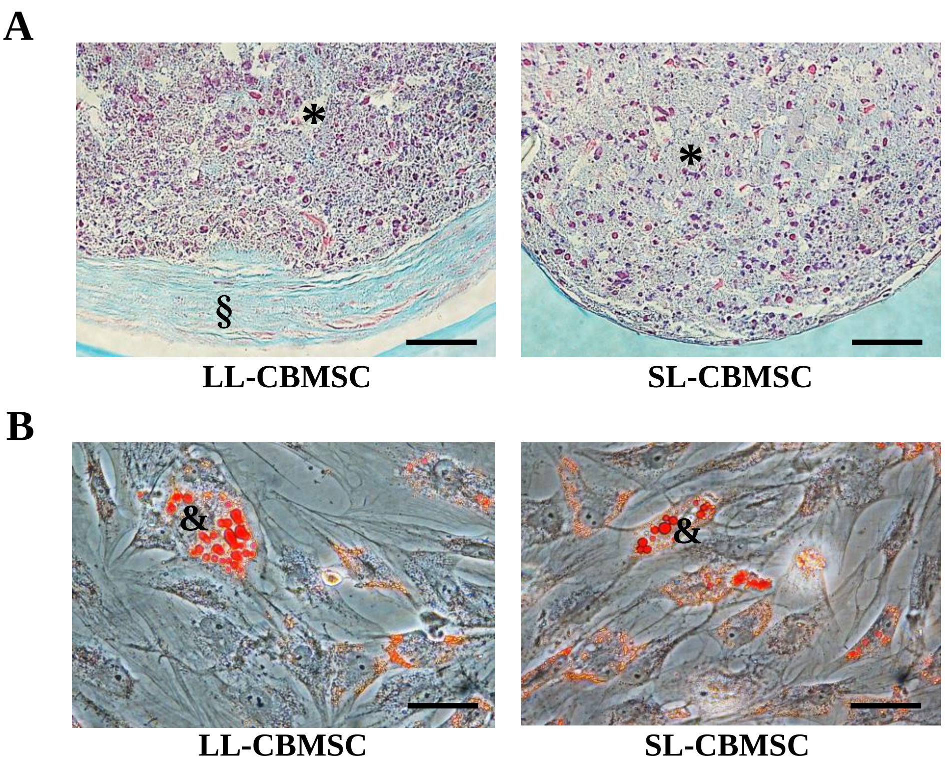

Supplementary figure 2. LL- and SL-CBMSC chondrogenic and adipogenic potential. Representative images of histological analysis of LL-CBMSC and SL-CBMSC chondrogenic cell pellets, stained by hematoxylin-eosin and alcian blue (A). Scale bar: 100 µm; * indicates undifferentiated cell pellet core, § indicates Alcian blue-positive chondromyxoid fibrotic tissue cuticle. Representative images of LL- and SL-CBMSC differentiated toward the adipogenic lineage, stained by Oil Red O (B). Scale bar: 50 µm; & indicates Oil Red O-positive lipid droplets.

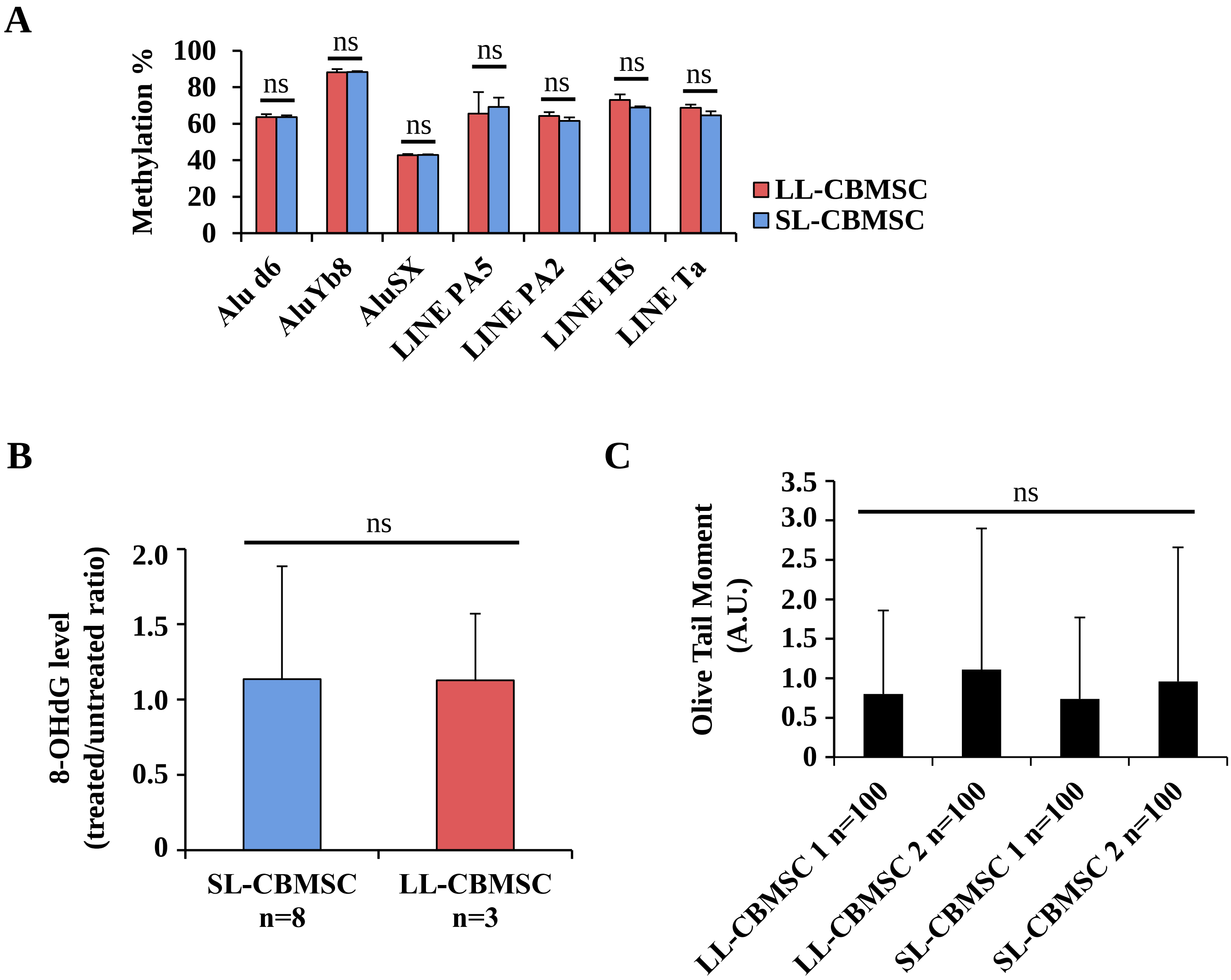

Supplementary figure 3. LL- and SL-CBMSC do not show significant difference in nuclear DNA global methylation and oxidative damage. Different LINE and SINE repeated sequences were analyzed as an estimation of global nuclear DNA methylation (A). Ns, not statistically significant (Student's t-test, LL-CBMSC vs. SL-CBMSC). The presence of 8-OHdG oxidative stress-associated DNA lesions on mitochondrial DNA was addressed by hOGG1 enzyme digestion and subsequent qPCR (B). Ns, not statistically significant (Student's t-test, LL-CBMSC vs. SL-CBMSC). Also single cell-gel electrophoresis and calculation of olive tail moment indicative of oxidative stress-associated genome fragility were performed (C). Ns, not statistically significant (one-way ANOVA followed by Holm-Sidak's post-hoc multiple comaprisons test). In all figures mean and standard deviation are represented.

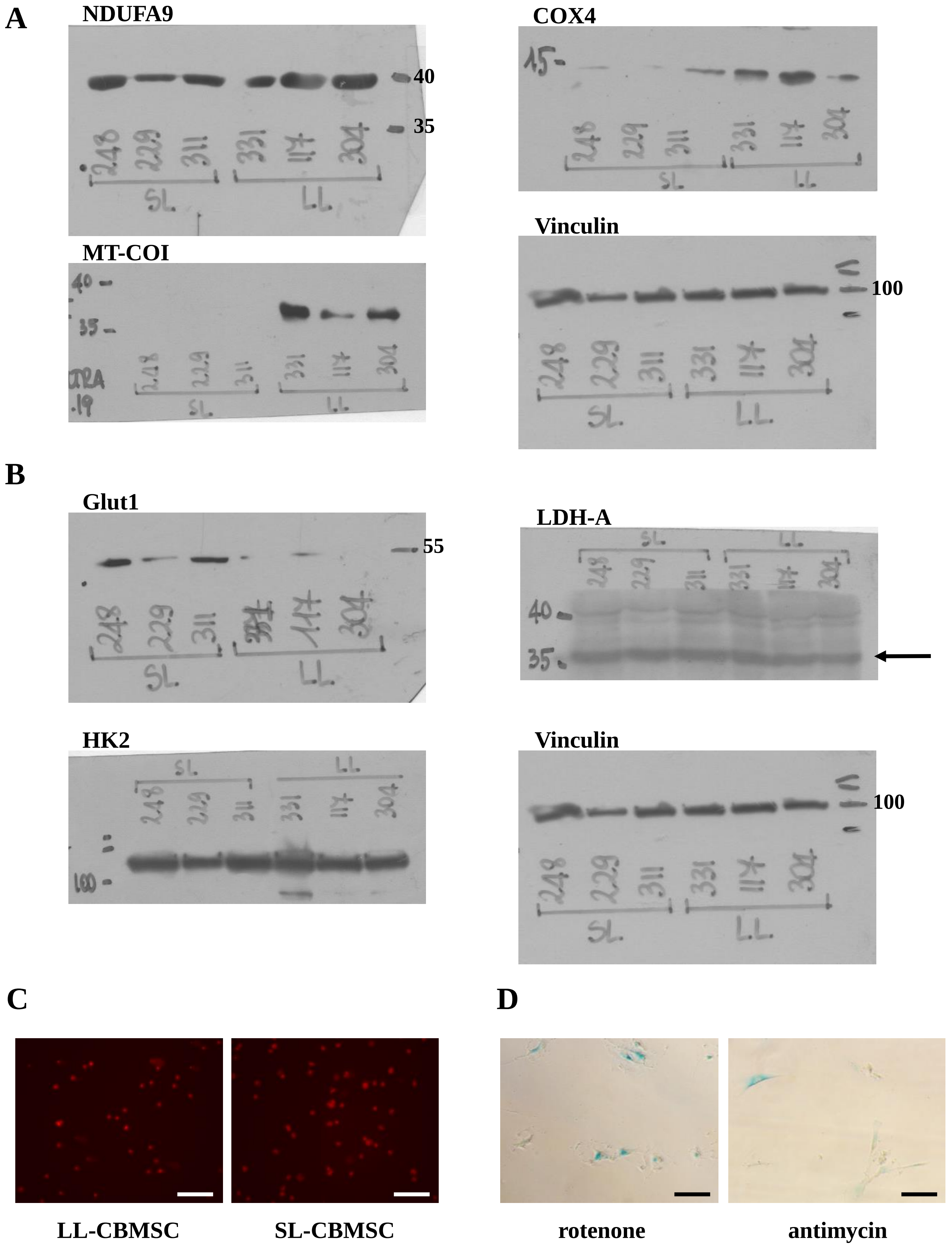

Supplementary figure 4. SL-CBMSC as compared to LL-CBMSC show a decrease of mitochondrial OXPHOS expression and an increase of Glut1 expression. Western blot analysis of the expression levels of the mitochondrial and glycolytic proteins. Vinculin was used to normalize the expression levels. Full scan images of immunoblots for main figures: A) mitochondrial proteins (main figure 3C); B) glycolytic proteins (main figure 4B). (C) Representative images of SCGE images for LL- and SL-CBMSC. Scale bar is 100 μm. (D) Representative images of β-galactosidase-positive LL-CBMSC after mitochondrial complex I (rotenone) or complex III (antimycin A) inhibition. Scale bar is 50 μm.

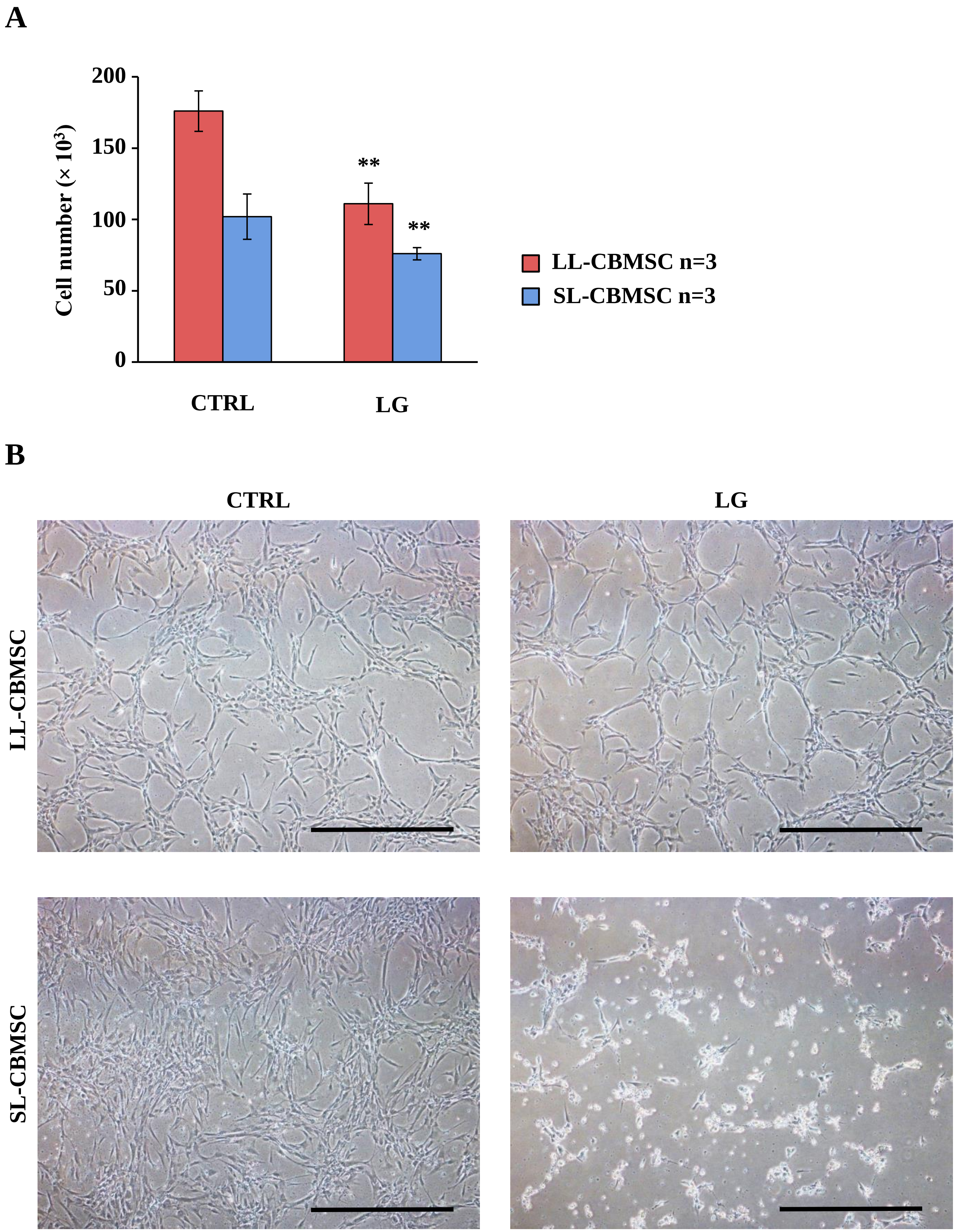

Supplementary figure 5. Glucose shortage induced proliferation arrest in both LL-and SL-CBMSC. (A) Cell number for LL- and SL-CBMSC after 48 hours of growth in standard conditions (CTRL) and in low glucose (LG) was assessed. $^{**}p<0.01$ (Mann-Whitney test, CTRL vs. LG). Mean and standard deviation are represented. (B) Representative images of LL- and SL-CBMSC in control or low glucose culture conditions. Scale bar is 1000 µm.

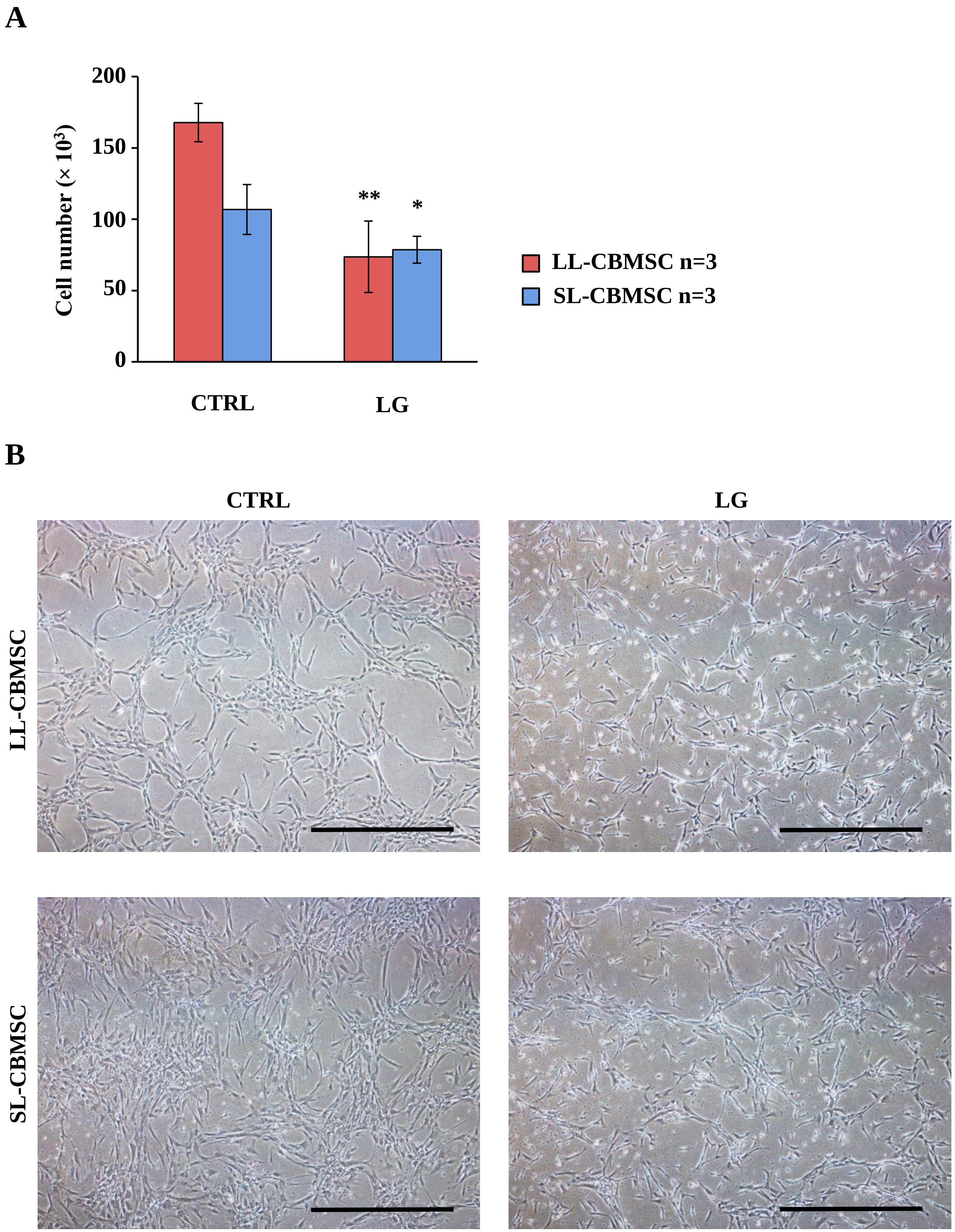

Supplementary figure 6. Glutamine shortage induced proliferation arrest in both LL- and SL-CBMSC. Cell number of LL- and SL-CBMSC after 48 hours of growth in standard conditions (CTRL) and in glutamine shortage (Low Gln) was addressed. *p<0.05, **p<0.01 (Mann-Whitney test, CTRL vs. LG). Mean and standard deviation are represented. (B) Representative images of LL- and SL-CBMSC in control or low glutamine culture conditions. Scale bar is 1000 μm.
